# A 3-year-old child with multiple superficial erosions and yellowish crusts on the scalp and seborrheic areas

**DOI:** 10.1016/j.jdcr.2024.01.034

**Published:** 2024-02-19

**Authors:** Leelawadee Techasatian, Napat Laoaroon, Kunanya Suwannaying, Patcharee Komwilaisak, Rattapon Uppala, Panthila Sitthikarnkha, Suchaorn Saengnipanthkul, Piti Ungarreevittaya, Suteeraporn Chaowattanapanit, Charoen Choonhakarn

**Affiliations:** a Pediatric Division, Faculty of Medicine, Khon Kaen University, Khon Kaen, Thailand; b Pathology Division, Faculty of Medicine, Khon Kaen University, Khon Kaen, Thailand; c Dermatology Division, Faculty of Medicine, Khon Kaen University, Khon Kaen, Thailand

**Keywords:** children, pemphigus foliaceus, rituximab

## Case presentation

**Fig 1:**
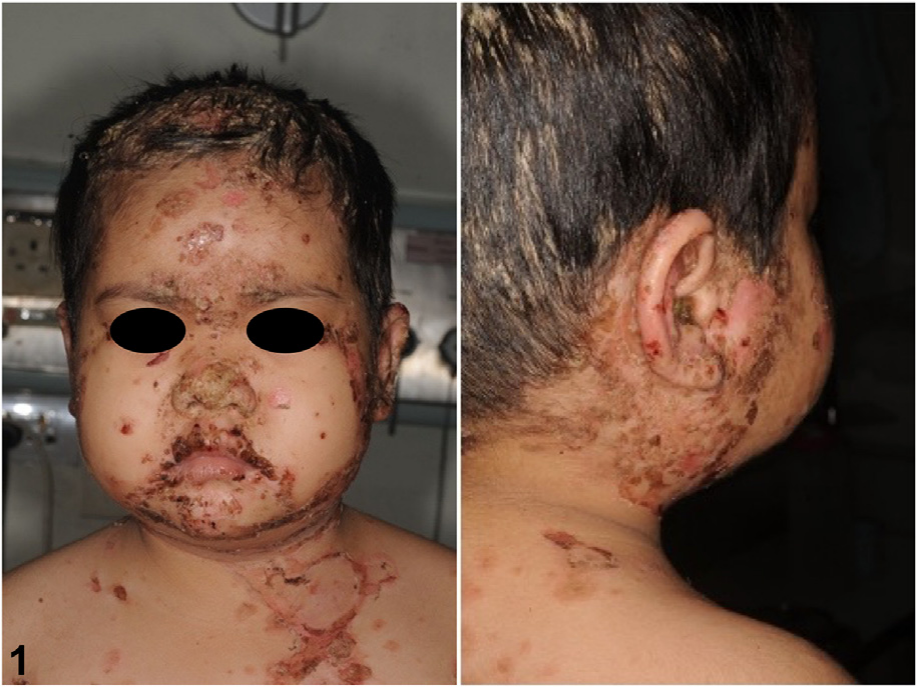

**Fig 2:**
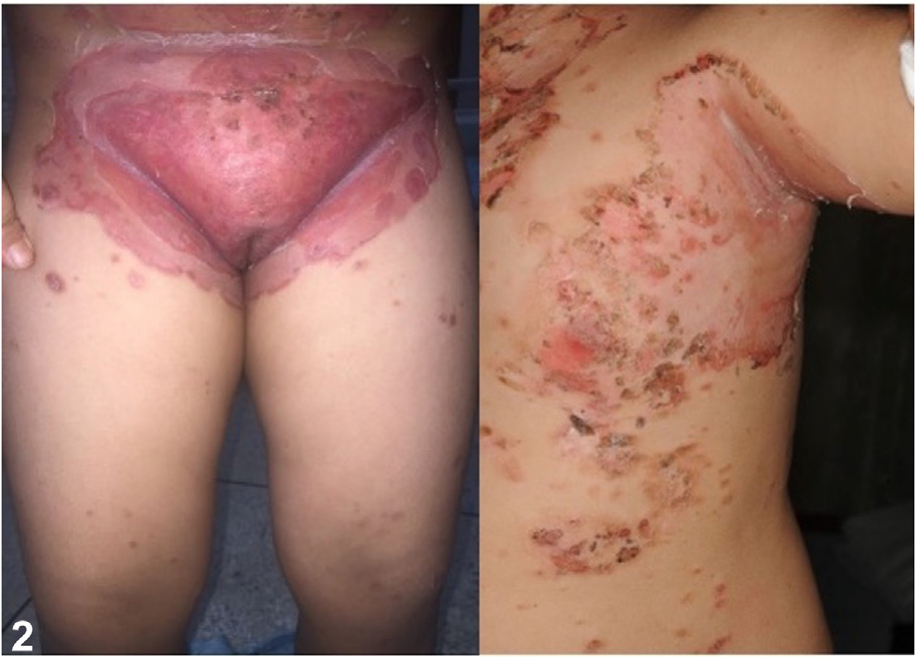

A three-year-old previously healthy girl with no underlying disease presented with multiple erythematous and yellowish plaques on face, scalp, and trunk for 2 months. Physical examination revealed scattered superficial yellowish, some with hemorrhagic crusts on erythematous patches on her scalp, face, nose, postauricular areas, trunk, back, buttocks, and groin region (Figs 1 and 2).

**Fig 3:**
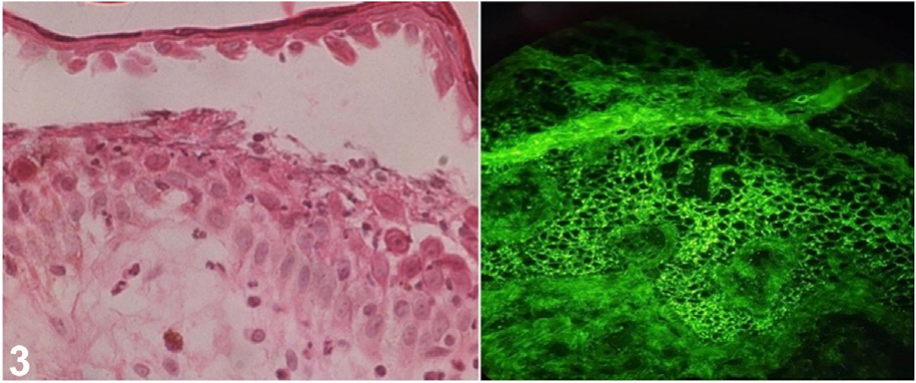

A skin biopsy was performed, histological examination revealed an intraepidermal blister with acantholytic cells. Direct immunofluorescence (DIF) was performed from peri-lesion to confirm the diagnosis, and it revealed IgG and C3 deposition in a granular pattern at the intercellular junction (Fig 3).

**Question 1: Based on clinical presentation and histopathology, what is the most likely diagnosis?**

A. Bullous impetigo
B. Inverse psoriasis
C. Pemphigus foliaceus (PF)
D. Langerhans cell histiocytosis (LCH)
E. Chronic bullous dermatosis of childhood (CBDC)

**Answers:**

A. Bullous impetigo – Incorrect. Bullous impetigo is a superficial bacterial skin infection. Although in this condition, antibodies also target DSg1 similar to PF, the clinical course is distinct. Bullous impetigo generally resolves in 1–2 weeks and responds favorably to systemic and topical antibiotics against Staphylococcus aureus or Streptococcus pyogenes bacteria. The organism should be apparent in the staining findings of the tissue.
B. Inverse psoriasis – Incorrect. The histopathology of psoriasis typically reveals several distinctive features including elongation of rete ridges and the presence of neutrophils in the epidermis, notably in the form of Munro's microabscesses. DIF is typically negative in psoriasis.
C. Pemphigus foliaceus (PF) – Correct. PF is a rare autoimmune bullous disorder in children. The cutaneous manifestations are often found on the face, scalp, axilla, intertriginous region, and in particular instances, the seborrheic distribution. Characteristics include crusted plaques and superficial erosions in a polycyclic pattern that can progress to erythroderma and exfoliative dermatitis. Histological examination revealed an intraepidermal blister with acantholytic cells within or adjacent to the granular layer. Dsg1 is being targeted and DIF revealed IgG and C3 deposition in a granular pattern at the intercellular junction confirming the diagnosis of pemphigus foliaceus.
D. Langerhans cell histiocytosis (LCH) – Incorrect. Even though the scalp, diaper area, and seborrheic distribution were conspicuous in this instance, histological testing confirmed the absence of Langerhans cells from the affected area, and LCH can be ruled out.
E. Chronic bullous dermatosis of childhood (CBDC) – Incorrect. The CBDC skin lesion is characterized by a central bulla surrounded by smaller curved or ovoid vesicles, a pattern that has given it various descriptive names, including "rosette," "string of pearls," and "cluster of jewels." The lower trunk, vulvar region, and upper thighs are the most typically affected areas in children. In CBDC, DIF should show linear IgA deposits along the basement membrane at the dermo-epidermal junction.

**Question 2: Which of the following is the pathophysiology of this skin condition?**

A. Toxin produces intradermal cleavage
B. Enzymatic defect in the heme biosynthetic pathway
C. Autoantibodies reactive against Ro and La antigens
D. Circulating autoantibodies directed against keratinocyte cell surfaces
E. Immune response to hemidesmosomal proteins within the dermal-epidermal junction

**Answers:**

A. Toxin produces intradermal cleavage – Incorrect. The pathogenesis of staphylococcal scalded skin syndrome (SSSS) is caused by an exfoliative toxin produced by Staphylococcus aureus. Bullae, or fluid-filled blisters, are caused by the epidermis splitting due to desmoglein-1 cleavage.
B. Enzymatic defect in the heme biosynthetic pathway – Incorrect. Enzymatic defect in the heme biosynthetic pathway is the pathophysiology of porphyria. Porphyrias are a group of rare genetic disorders that affect the heme biosynthetic pathway, leading to an accumulation of heme precursors known as porphyrins.
C. Autoantibodies reactive against Ro and La antigens – Incorrect. Autoantibodies reactive against Ro and La antigens are the pathophysiology of neonatal lupus erythematosus.
D. Circulating autoantibodies directed against keratinocyte cell surfaces – Correct. Circulating autoantibodies target specific proteins or structures on the surface of keratinocytes, leading to the disruption of cell adhesion and blister formation which is the pathophysiology of pemphigus foliaceus (antibodies target Dsg 1) and pemphigus vulgaris (antibodies target Dsg 1 and Dsg3).
E. Immune response to hemidesmosomal proteins within the dermal-epidermal junction – Incorrect. Autoantibodies against hemidesmosomal proteins (BP180 and BP230) within the dermal-epidermal junction lead to neutrophil chemotaxis and degradation of the basement membrane zone is the pathophysiology of bullous pemphigoid disease.

**Question 3: What are the treatments of this skin condition?**

A. Doxycycline
B. Itraconazole
C. Griseofulvin
D. Cloxacillin
E. Rituximab

**Answers:**

A. Doxycycline – Incorrect. Doxycycline is an antibiotic used in the treatment of infections caused by bacteria.
B. Itraconazole – Incorrect. Itraconazole is an antifungal drug that specifically treats fungal infections.
C. Griseofulvin – Incorrect. Griseofulvin is an antifungal medicine specifically used to treat fungal infections.
D. Cloxacillin – Incorrect. Cloxacillin is specifically used to treat gram-positive bacterial infections.
E. Rituximab – Correct. Rituximab, corticosteroids, methotrexate, and cyclosporine have all been used to treat pemphigus foliaceus. Systemic corticosteroids are the mainstay of therapy, however other systemic immunosuppressive medications such as cyclosporine and methotrexate have been used as steroid-sparing medications. Rituximab was recently approved in Europe and the United States as a first-line therapy for moderate to severe pemphigus vulgaris. A recent guideline recommends the use of rituximab as a major therapeutic approach in adults with pemphigus disease. The following first-line therapies of moderate and severe pemphigus in adults are recommended to start rituximab (two infusions of 1 g two weeks apart) associated with systemic corticosteroids (prednisone 1 mg/kg/ day) with a progressive tapering in order to stop corticosteroids after 6 months. There is very little data on therapy in children. The presenting case was treated with rituximab (375 mg/m^2^/dose) for two courses with three weeks apart. To control secondary bacterial skin infection, wound scrub, wound dressing, and intravenous antibiotics were administered. After two courses of rituximab, cutaneous lesions improved, and overall lesions were subsided. This case report details the effective use of rituximab in treating PF in a three-year-old girl, resulting in a positive outcome and a safe treatment profile.^1^, ^2^, ^3^, ^4^, ^5^

## Conflicts of interest

None disclosed.
